# Systemic cytokines and GlycA discriminate disease status and predict corticosteroid response in HTLV-1-associated neuroinflammation

**DOI:** 10.1186/s12974-022-02658-w

**Published:** 2022-12-08

**Authors:** Tatiane Assone, Soraya Maria Menezes, Fernanda de Toledo Gonçalves, Victor Angelo Folgosi, Gabriela da Silva Prates, Tim Dierckx, Marcos Braz, Jerusa Smid, Michel E. Haziot, Rosa M. N. Marcusso, Flávia E. Dahy, Evelien Vanderlinden, Sandra Claes, Dominique Schols, Roberta Bruhn, Edward L. Murphy, Augusto César Penalva de Oliveira, Dirk Daelemans, Jurgen Vercauteren, Jorge Casseb, Johan Van Weyenbergh

**Affiliations:** 1 Laboratory of Medical Investigation LIM 56, Division of Dermatology and Institute of Tropical Medicine of Sao Paulo, Medical School, University of São Paulo, São Paulo, SP Brazil; 2 Laboratory of Immunohematology and Forensic Hematology-LIM40, Department of Forensic Medicine, Medical Ethics, Social Medicine and Work, University of São Paulo Medical School, São Paulo, Brazil; 3 Laboratory of Clinical and Epidemiological Virology, Department of Microbiology, Immunology and Transplantation, Rega Institute for Medical Research, KU Leuven, Leuven, Belgium; 4 Programa de Pós-Graduação em Ciências da Saúde, Faculdade de Medicina da Bahia, Universidade Federal da Bahia, Salvador, Bahia Brazil; 5 Institute of Infectious Diseases “Emilio Ribas” (IIER) de São Paulo, São Paulo, SP Brazil; 6 Laboratory of Virology and Chemotherapy, Department of Microbiology, Immunology and Transplantation, Rega Institute for Medical Research, KU Leuven, Leuven, Belgium; 7 Vitalant Research Institute, San Francisco, CA USA; 8 University of California San Francisco, San Francisco, CA USA

**Keywords:** HTLV-1, HAM/TSP, Cytokines, Inflammation, Corticosteroids

## Abstract

**Background:**

HTLV-1-Associated Myelopathy/Tropical Spastic Paraparesis (HAM/TSP) is an incapacitating neuroinflammatory disorder for which no disease-modifying therapy is available, but corticosteroids provide some clinical benefit. Although HAM/TSP pathogenesis is not fully elucidated, older age, female sex and higher proviral load are established risk factors. We investigated systemic cytokines and a novel chronic inflammatory marker, GlycA, as possible biomarkers of immunopathogenesis and therapeutic response in HAM/TSP, and examined their interaction with established risk factors.

**Patients and methods:**

We recruited 110 People living with HTLV-1 (PLHTLV-1, 67 asymptomatic individuals and 43 HAM/TSP patients) with a total of 946 person-years of clinical follow-up. Plasma cytokine levels (IL-2, IL-4, IL-6, IL-10, IL-17A, IFN-γ, TNF) and GlycA were quantified by Cytometric Bead Array and ^1^NMR, respectively. Cytokine signaling and prednisolone response were validated in an independent cohort by nCounter digital transcriptomics. We used multivariable regression, machine learning algorithms and Bayesian network learning for biomarker identification.

**Results:**

We found that systemic IL-6 was positively correlated with both age (*r* = 0.50, *p* < 0.001) and GlycA (*r* = 0.45, *p* = 0.00049) in asymptomatics, revealing an ‘inflammaging” signature which was absent in HAM/TSP. GlycA levels were higher in women (*p* = 0.0069), but cytokine levels did not differ between the sexes. IFN-γ (*p* = 0.007) and IL-17A (*p* = 0.0001) levels were increased in untreated HAM/TSP Multivariable logistic regression identified IL-17A and proviral load as independent determinants of clinical status, resulting in modest accuracy of predicting HAM/TSP status (64.1%), while a machine learning-derived decision tree classified HAM/TSP patients with 90.7% accuracy. Pre-treatment GlycA and TNF levels significantly predicted clinical worsening (measured by Osame Motor Disability Scale), independent of proviral load. In addition, a poor prednisolone response was significantly correlated with higher post-treatment IFN-γ levels. Likewise, a transcriptomic IFN signaling score, significantly correlated with previously proposed HAM/TSP biomarkers (*CASP5/CXCL10/FCGR1A/STAT1*), was efficiently blunted by in vitro prednisolone treatment of PBMC from PLHTLV-1 and incident HAM/TSP.

**Conclusions:**

An age-related increase in systemic IL-6/GlycA levels reveals inflammaging in PLHTLV-1, in the absence of neurological disease. IFN-γ and IL-17A are biomarkers of untreated HAM/TSP, while pre-treatment GlycA and TNF predict therapeutic response to prednisolone pulse therapy, paving the way for a precision medicine approach in HAM/TSP.

**Supplementary Information:**

The online version contains supplementary material available at 10.1186/s12974-022-02658-w.

## Introduction

Human T-cell Lymphotropic Virus type-1 (HTLV-1) is unique as it is both oncogenic [1, 2] and capable of triggering HTLV-1-Associated Myelopathy/Tropical Spastic Paraparesis (HAM/TSP) and other inflammatory diseases [3–5]. Worldwide, 10 million people are estimated to be living with HTLV-1 (PLHTLV-1) [3], of which 1–2% develop HAM/TSP, an incapacitating, progressive neuroinflammatory disorder [4, 5].

Currently, no disease-modifying therapy is available for HAM/TSP but corticosteroids and other immunomodulators (IFN-α, methotrexate, cyclosporin) provide some clinical benefit [4–6]. Moreover, we recently demonstrated HAM/TSP is an independent predictor for early mortality among PLHTLV-1 [7]. As such, validated biomarkers to predict and/or monitor disease progression for PLHTLV-1 and therapeutic outcome in HAM/TSP patients are direly needed [8].

Acute inflammation is routinely measured by systemic cytokines, while glycoprotein acetylation (GlycA) represents a novel marker for chronic inflammation, which reliably predicts long-term outcomes of inflammatory and infectious diseases in patient cohorts or large prospective population studies [9–12]. Up to now, GlycA has not been explored in HTLV-1 infection or HAM/TSP. Lifelong chronic infection with other latent viruses (CMV and HIV) causes long-term activation of the immune system over time, contributing to inflammaging [13]. Although extensively described in people living with HIV-1 [14, 15], data on inflammaging are lacking in PLHTLV-1. Therefore, we investigated pro- and anti-inflammatory cytokines and GlycA as possible biomarkers of inflammaging, immunopathogenesis and therapeutic response in HAM/TSP, using unique samples from a large cohort of PLHTLV-1 with an exceptionally long clinical follow-up (range 2–20 years).

## Patients and methods

### Cohort characteristics, patient recruitment and sampling strategy

From and ongoing open cohort study researching the natural history of HTLV-1 infection [7], we selected all first available plasma samples (closest to recruitment into the cohort) from patients with definite HAM/TSP before treatment, as well as age- and gender-matched first available samples (closest to recruitment into the cohort) of PLHTLV-1 who were neurologically asymptomatic (AS) during clinical follow-up (study period: August 1997 to December 2019). All volunteers underwent serological screening for HTLV-1 at the “Emilio Ribas” Institute of Infectious Diseases, using GOLD ELISA HTLV-1/2 (Diasorin, UK), followed by confirmation with Western Blot (MP Diagnostics, HTLV Blot 2.4®) and in-house nested PCR [16]. Paired follow-up samples were obtained after methylprednisolone pulse therapy for 38 HAM/TSP patients (several cycles per patient, total duration ranged from 1.5 to 18.6 years). Blood samples were collected in K_3_-EDTA (0.054 ml/tube), plasma was separated by centrifugation (15 min, 2500 rpm) and PBMC were purified by Ficoll density gradient centrifugation (GE Healthcare Life, USA). Cells were washed with saline solution, the cell number was adjusted to 10^6^ cells, followed by storage (as “dry pellet”) at − 80 °C. DNA was extracted using a commercial kit (Illustra Tissue and Cells Genomic Prep Mini Spin kit, Fairfield, CA) according to the manufacturer’s instructions, and stored at − 80 °C.

### Quantification of HTLV-1 proviral load (PVL)

HTLV-1 proviral load was quantified by real-time PCR, using primers and probes targeting the HTLV-1 *pol* gene, with the human albumin gene as internal reference, as described previously [16]. All samples were analyzed in duplicate, and results expressed as HTLV-1 DNA copies/10^6^ PBMCs.

### Neurological evaluation, HAM/TSP diagnosis and treatment

PLHTLV-1 were classified in two groups according to their neurological status: 67 asymptomatic HTLV-1-infected individuals and 43 HAM/TSP patients, of which 38 provided paired samples after treatment with methylprednisolone (1 g intravenously, every 45 days). HAM/TSP diagnostic criteria was based on recommendations from an international consortium [17]. Clinical evaluation and a standardized screening neurological examination were performed by a board-certified neurologist, blinded for HTLV-1 clinical status for all subjects. For clinical follow-up, the Osame Motor Disability Scale was used, ranging from 0 (no walking or running disabilities) to 13 (cannot even move toes). Methylprednisolone pulse therapy was offered to all HAM/TSP patients as first-line treatment, except for those with diabetes and/or urinary infection, the latter were excluded from this study.

### Plasma cytokine levels

Concentrations of plasma (undiluted) cytokines were measured using the CBA (Cytometric Bead Array, BD Biosciences) Human Th1/Th2/Th17 Cytokine Kit, including Interleukin-2 (IL-2), Interleukin-4 (IL-4), Interleukin-6 (IL-6), Interleukin-10 (IL-10), Tumor Necrosis Factor (TNF), Interferon-γ (IFN-γ), and Interleukin-17A (IL-17A), in accordance with the manufacturer’s instructions.

### Plasma CXCL10 levels

An additional biomarker, the chemokine CXCL10 (also known as IP-10) was quantified with the Bio-Plex Pro Human Chemokine IP-10/CXCL10 kit (Bio-Rad), following the manufacturer’s instructions [18].

### GlycA (glycoprotein acetyl) quantification in plasma

GlycA concentration was quantified using the Nightingale Health Ltd. high-throughput metabolomics platform (Helsinki, Finland), as previously described [9]. Briefly, a ^1^H-NMR spectrum is taken from 350 μl of plasma, with the area under the peak measured at approximately 2 ppm quantifying signal originating from N-acetyl sugar groups present on acute phase glycoproteins (α-1-acid glycoprotein, α-1-antitrypsin, α-1-antichymotryspin, haptoglobin, transferrin).

### Data collection and quality control

Data entry in the electronic database RedCap [19] was performed by two administrative assistants, and subsequently checked by the first and last author.

### Ethical issues

The Ethical Board of “Instituto de Infectologia Emilio Ribas”, Sao Paulo-Brazil, approved the protocol (Number 07688818.2.1001.0061). Signed informed consent was obtained from all participants prior to study inclusion.

### Digital transcriptomics and biological pathway analysis

Digital transcriptomic analysis (nCounter, Nanostring Technologies) and biological pathway analysis of in vitro prednisolone response was performed as previously described [20, 21], using the Myeloid/Innate Immunity Panel, consisting of > 750 host genes, as well as customized HTLV-1 Hbz and Tax probes. Specific cytokine signaling scores were calculated as z-scores of the geometric mean of a set of curated transcripts (nSolver software) for each cytokine or cytokine family (IL-10, IFN, IL-17, IL4/IL-13, IL-2 family). PBMCs were obtained from an independent, previously characterized cohort of PLHTLV-1 (4 asymptomatic, 4 HAM/TSP patients), as well as age-, gender- and ethnicity-matched healthy controls (*n* = 4) from the HOST study [22]. PBMCs were cultured in RPMI supplemented with 10% FCS, in the absence or presence of 10 µg/ml of prednisolone (Sigma) for a short time period (36 h), as determined by our previous studies [21, 23–26].

### Statistical analysis, machine learning and Bayesian network analysis

Statistical analysis was performed using XLStat and GraphPad Prism version 9, San Diego, CA). Logistic regression and non-parametric statistical tests (Mann–Whitney, Wilcoxon tests, Spearman correlation) were used, except for Maximal Osame Motor Disability Score (which followed normal distribution, ANOVA), with Bonferroni correction for multiple comparisons as indicated in the text. For prednisolone response in vitro, one sample t test was used to compare groups to the mean value of incident HAM/TSP, since numbers (4 in each group) were too small to apply generalized linear mixed models. Machine learning algorithms (attribute selection, J48 and PART decision trees) were applied using Weka (version 3.8.4). Bayesian network analysis was performed as previously described [27].

## Results

### Age and gender differentially affect cytokines and GlycA in people living with HTLV-1, independent of proviral load

**Fig. 1:**
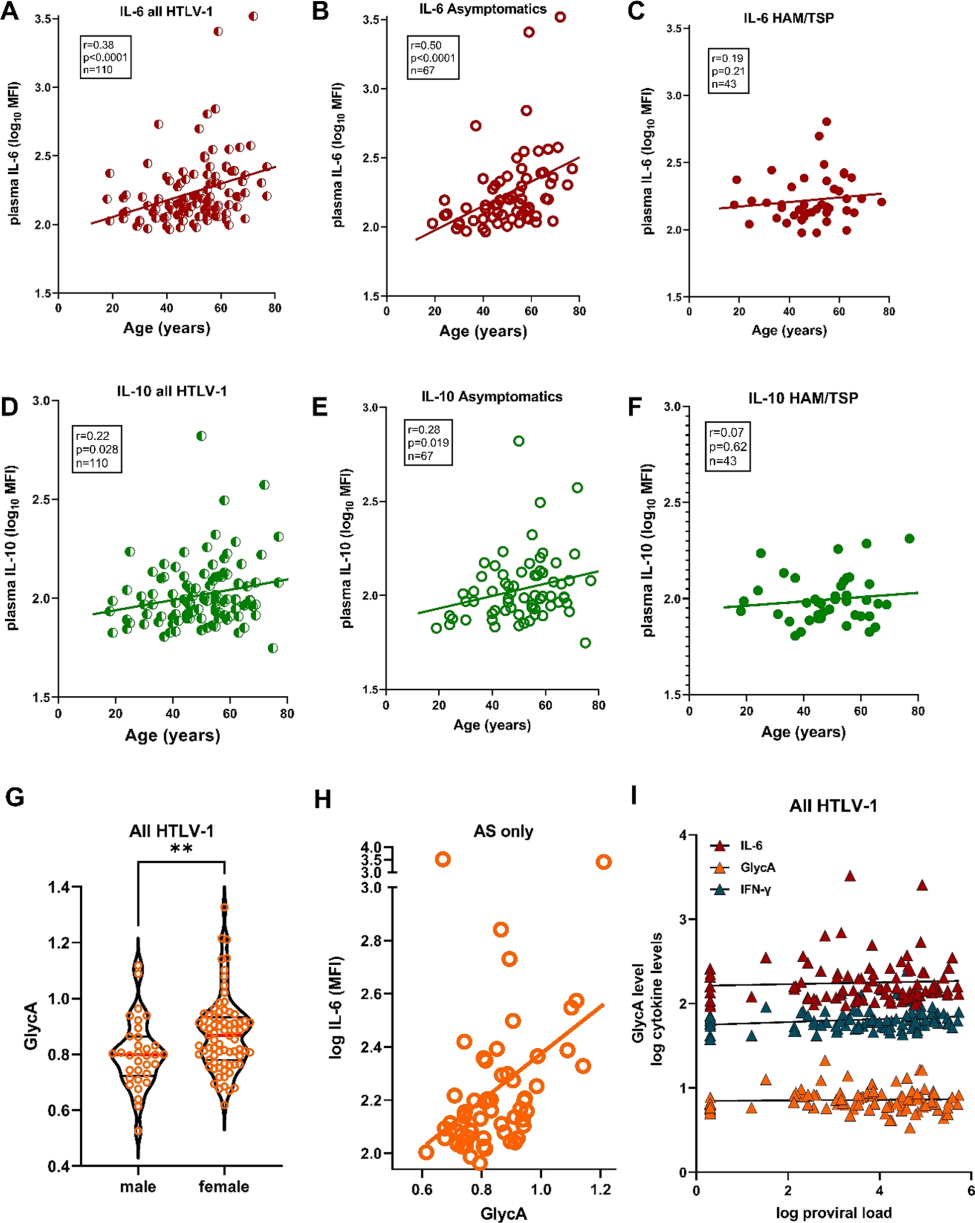
Age and gender differentially affect cytokines and chronic inflammation marker GlycA in people living with HTLV-1, independent of proviral load. **A** IL-6 levels are significantly correlated with age at sampling in all PLHTLV-1, driven by the strong correlation in asymptomatic individuals (AS) (**B**), which is absent in HAM/TSP patients (**C**). **D** IL-10 levels are significantly correlated with age at sampling in all PLHTLV-1, driven by the strong correlation in AS (**E**), which is absent in HAM/TSP patients (**F**). **G** GlycA levels are significantly higher in female PLHTLV-1 (*p* = 0.0069, Mann–Whitney test). **H** GlycA levels are positively correlated with IL-6 levels in AS only (*p* = 0.00049, *r* = 0.45). **I** Proviral load is not significantly correlated with IL-6 (*ρ* = 0.07, *p* = 0.45) or GlycA levels (*ρ* = − 0.06, *p* = 0.72) in PLHTLV-1, while a tendency is observed for IFN-γ (*ρ* = 0.20, *p* = 0.054). Correlation is determined using Spearman’s method, uncorrected *p* values are reported, significant correlations of IL-6 with age and with GlycA were robust to correction for multiple testing (Bonferroni *p* < 0.05)

As shown in Table 1, HAM/TSP patients were age- and gender-matched to HTLV-1-infected controls without neurological symptoms (asymptomatics, AS), while proviral load was increased in HAM/TSP, as expected [4, 5]. Since age and gender are major determinants of HAM/TSP pathogenesis, as disease onset usually occurs after several decades and women are more affected [4, 5], we investigated if cytokines or GlycA were linked to these demographics, in the absence or presence of neuroinflammation. Biological sex did not influence systemic cytokine levels (data not shown), whereas both IL-6 (Spearman’s *r* = 0.36, *p* = 0.00018) and IL-10 (*r* = 0.22, *p* = 0.021) were positively correlated with age in PLHTLV-1 (Fig. 1A–D). Surprisingly, this age-dependent cytokine increase was specific to AS (*r* = 0.50, *p* < 0.0001, Fig. 1B–E), and absent in HAM/TSP (Fig. 1C–F). In contrast, chronic inflammation marker GlycA did not correlate with age (*p* = 0.12), but was higher in females (*p* = 0.0069, Fig. 1G). Among all cytokines, only IL-6 was significantly correlated with GlycA in AS (Fig. 1H, *p*=0.00049, *r* = 0.45) but not HAM/TSP patients (*p* = 0.16), revealing an “inflammaging” signature which was surprisingly limited to PLHTLV-1 without neuroinflammatory disease. Correlations of IL-6 with age and with GlycA remained significant after Bonferroni correction for multiple testing, while IL-10 correlation with age was not. Of note, none of the cytokines nor GlycA were significantly correlated with proviral load (Fig. 1I and not shown), but a tendency was observed for IFN-γ (Fig. 1I, *r*= 0.20, *p* = 0.054).

**Table 1:** Principal characteristics of study participants (AS vs. HAM/TSP)

Variables	Asymptomatics (*n* = 67)	HAM/TSP (*n* = 43)	*P* value^a^
Gender n (%)			0.24
Male	20 (30.8)	14 (31.1)	
Female	47 (68.2)	29 (68.9)	
Age (years) in entry of cohort^b^			
Median (± SD)	51 (13)	48 (13)	0.40
Time Follow-up (years)			
Median (± SD)	8.6 (5.7)	8.6 (3.7)	0.37
Range (min–max)	1.5–20.8	1.5–18.6	
HTLV-1 Proviral Load^c^			
Median (95% CI)	3845 (839–18,953)	28,184 (14,464–53,864)	0.0007

^a^Statistical tests: chi-square for categorical variables, Mann–Whitney test for numerical variables

^b^Age missing for 1 HAM/TSP

^c^HTLV-1 DNA copies/10^6^ PBMCs, proviral load missing for 10 AS and 4 HAM/TSP

### Pro-inflammatory cytokines IFN-γ and IL-17A are biomarkers of untreated HAM/TSP

**Fig. 2:**
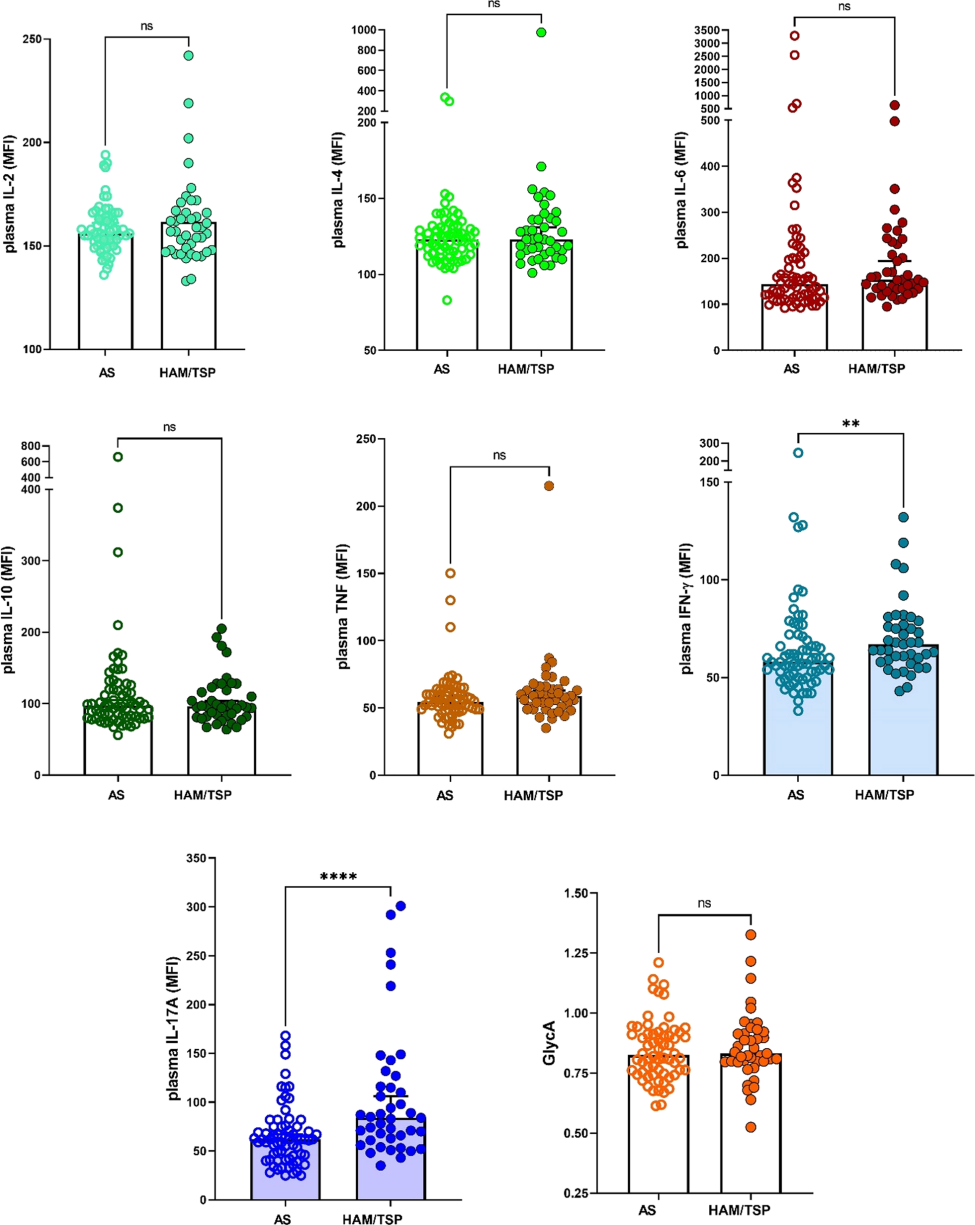
HAM/TSP disease status is characterized by increased IFN-γ and IL-17A. Among all cytokines tested, only IFN-γ (*p* = 0.0054) and IL-17A (*p* < 0.0001) levels in HAM/TSP patients (*n* = 43) were significantly different (Mann–Whitney test), as compared to asymptomatic individuals (AS, *n* = 67), both associations were robust to correction for multiple testing (Bonferroni *p* < 0.05)

**Fig. 3:**
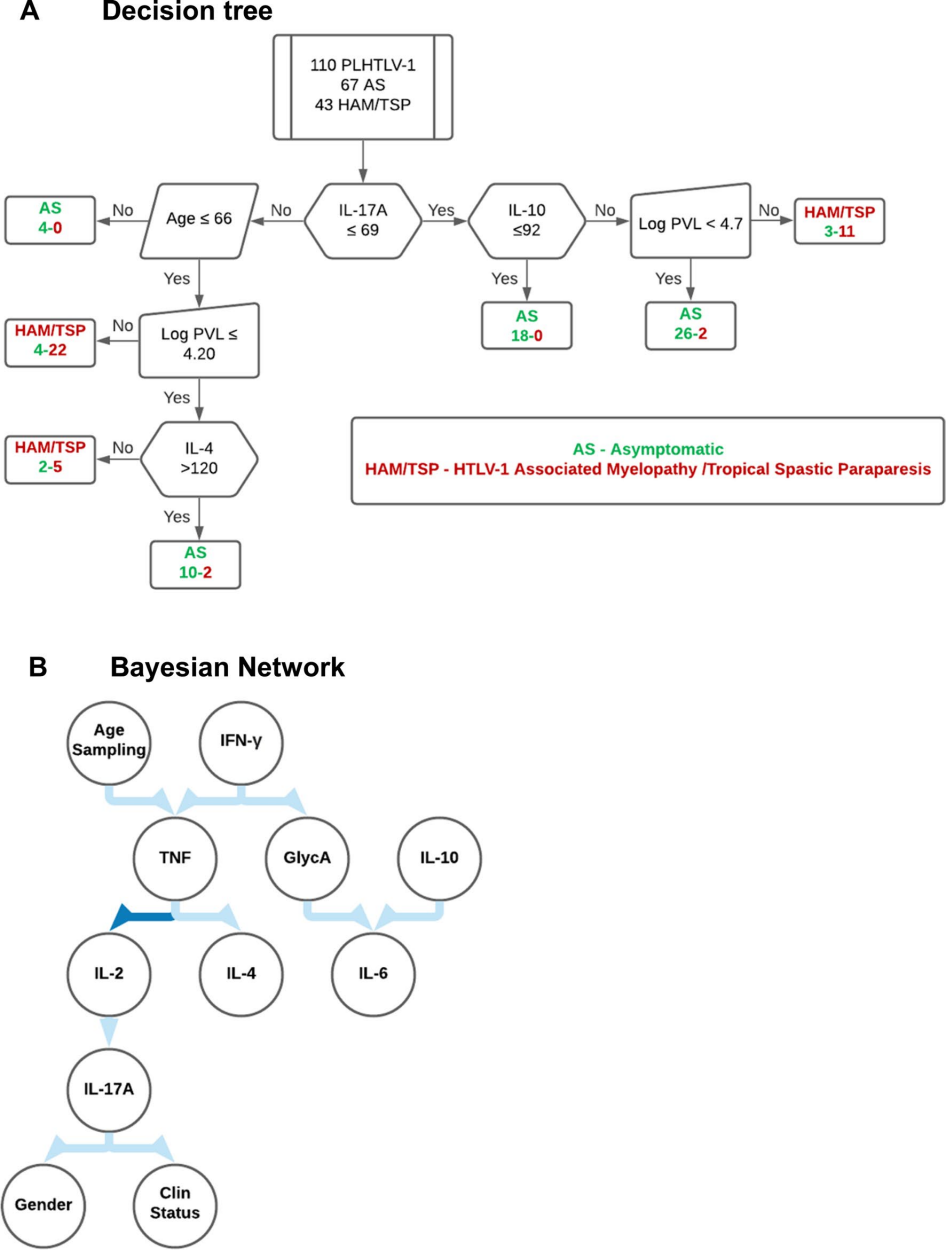
Machine Learning and Bayesian Network confirm IL-17A as central cytokine in HAM/TSP disease status. **A** Machine learning-derived decision tree discriminating AS from HAM/TSP patients (J48 pruned tree) with 90% accuracy (97 out of 110 PLHTLV-1 correctly classified, of which 61/67 AS and 38/43 HAM/TSP patients, ROC AUC 0.87, Kappa statistic 0.79). **B** Bayesian Network representing the strongest associations between cytokines, GlycA, clinical and demographic data (strength of arcs as defined previously [19]: dark blue 100×, light blue 10×). Proviral load was not significantly associated with the other parameters and hence not shown

When comparing cytokine levels between clinical groups, we observed a significant increase in IFN-γ (*p* = 0.007) and IL-17A (*p* = 0.0001) in HAM/TSP patients, as compared to AS, while other cytokines and GlycA did not differ (Fig. 2). Using logistic regression (detailed in Additional file 1: Table S1), we found that IL-17A and proviral load were independently associated with clinical status, consistent with a weak correlation between IFN-γ and proviral load (Fig. 1I). However, logistic regression resulted in low classification accuracy for HAM/TSP patients, as only 25/39 (64.1%) were correctly classified, in contrast to 49/57 (86.0%) of correctly predicted AS (ROC AUC 0.85). Therefore, we used machine learning algorithms to improve classification, which revealed a decision tree (Fig. 3A) classifying 39/43 HAM/TSP and 58/67 AS, respectively, with 90.7% and 86.6% accuracy (ROC AUC 0.87). Among the first branches in this decision tree are IL-17A and IL-10, confirming previous findings in a UK cohort [28].

Bayesian networks are graphical models that are widely used in systems biology to depict genes/proteins as nodes and connections between genes as edges, based on both linear and non-linear associations between the nodes. Thus, network connections in Bayesian networks have been demonstrated to accurately capture the functional and mechanistic relationship of genes/proteins that can inform on disease mechanisms and help define composite sets of molecular markers [29–31]. Therefore, we used Bayesian network learning to identify direct vs. indirect associations between cytokines, GlycA, clinical and demographic data, similar to previous analysis in HTLV-1-associated leukemia [27]. As shown in Fig. 3B, only IL-17A was directly connected to clinical status, while all other cytokines were ‘upstream’ of IL-17A. This Bayesian network revealed a direct link between GlycA and IL-6, whereas the observed correlation between age and IL-6 (Fig. 1A, B) appears dependent on TNF and IFN-γ, the latter directly influencing GlycA. IFN-γ was found upstream of all other cytokines and, consequently, of disease status, which underscores the previously identified IFN gene signature in HAM/TSP [32]. Of interest, in this unsupervised model, proviral load was not significantly associated with the other parameters and hence absent from the network.

### Systemic cytokines and GlycA are biomarkers of corticosteroid therapeutic response in HAM/TSP

**Fig. 4:**
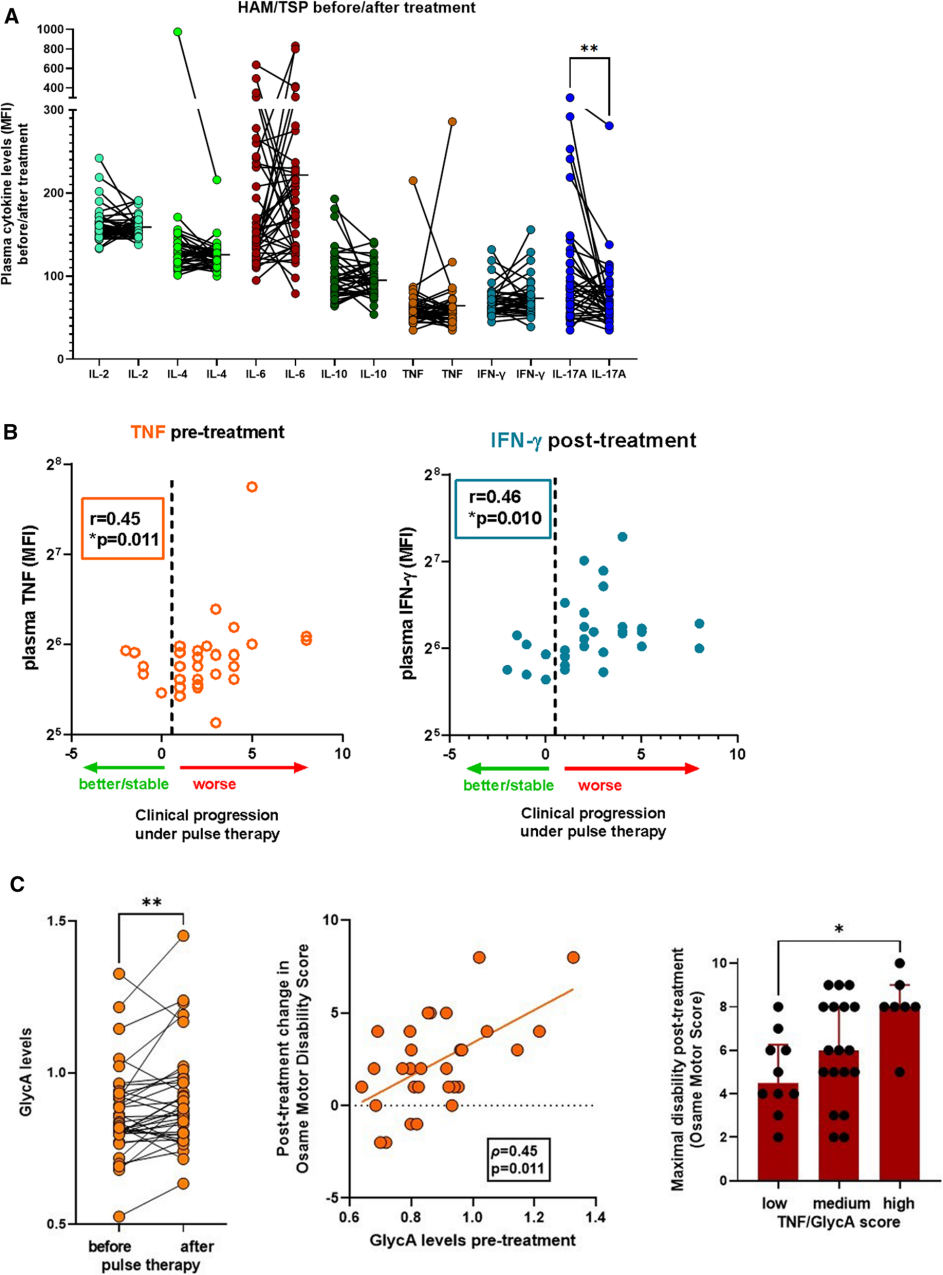
Cytokines and GlycA predict therapeutic success vs. failure of methylprednisolone pulse therapy in HAM/TSP patients. **A** Among all plasma cytokines measured in HAM/TSP patients, only IL-17A significantly decreases after pulse therapy with intravenous methylprednisolone (Wilcoxon test *p* = 0.003, Bonferroni correction *p* < 0.05). **B** Pre-treatment TNF (left panel) and post-treatment IFN-γ (right panel) are correlated with the magnitude of clinical worsening after methylprednisolone pulse therapy (Spearman correlation), measured as quantitative changes in Osame Motor Disability Score. **C** GlycA levels are significantly increased after methylprednisolone pulse therapy (*p* = 0.0087, Wilcoxon test, left panel). Pre-treatment GlycA levels are correlated with the magnitude of clinical worsening after methylprednisolone pulse therapy (Spearman correlation, middle panel). A higher TNF/GlycA score (0.025*TNF + 9.18*GlycA − 7.28) predicts worse Osame Motor Disability Score after methylprednisolone pulse therapy (right panel, ANOVA with Bonferroni post-test, *p = 0.010)

Next, we investigated if cytokines and GlycA might be candidate biomarkers for therapeutic response in HAM/TSP patients. In this cohort, all eligible patients were uniformly treated with intravenous methylprednisolone pulse therapy, which allowed unbiased comparisons before and after treatment. Patients with > 1 year pulse therapy follow-up were classified as responders (*n* = 13) and non-responders (*n* = 25), based on changes in Osame Motor Disability Score (decrease or ≤ 1: responders, increase > 1: non-responders). All patients significantly decreased IL-17A levels after treatment (Fig. 4A, *p*= 0.003, Wilcoxon test), while strong variability but no directionality was observed for any other cytokine. However, lower IFN-γ levels after treatment were correlated with better clinical response to corticosteroid pulse therapy (Fig. 4B, *p*= 0.010). In addition, pre-treatment TNF levels were significantly associated with therapeutic outcome (Fig. 4B, *p*= 0.011). In contrast to all cytokines, GlycA levels were significantly increased (Fig. 4C left panel, *p* = 0.0087, Wilcoxon test). Moreover, pre-treatment GlycA levels were able to predict therapeutic response, (measured by quantitative changes in Osame Motor Disability Score), either by itself (Fig. 4C, left panel) or as a combined TNF/GlycA score (Fig. 4C, right panel). Multivariate linear regression (Additional file 1:Table S2) confirmed both TNF and GlycA as independent predictors of disability progression, while age, gender and proviral load were not. Since disability progression was measured over a long period of time (range 1.5–18.6 years), including several cycles of pulse therapy for most patients, therapeutic response to corticosteroids cannot be unequivocally distinguished from the natural history of HAM/TSP, i.e., disease progression over time. Therefore, we calculated disease progression rate for both pulse time and total time of follow-up, as well as disease duration (time since onset of neurological symptoms). As shown in Additional file 1: Fig. S1A, only disease progression rate calculated as δOsame (quantitative changes in Osame Motor Disability Score) over total pulse time, but not over time of follow-up at sampling, was significantly correlated with pre-treatment GlycA levels (*r* = 0.42, *p* = 0.024, *n* = 29). Neither maximal disease progression over the total disease duration (Additional file 1: Fig. S1A), nor age of onset or age at entry in the cohort were significantly correlated with GlycA levels (Additional file 1: Fig. S1B) or TNF levels (data not shown). In addition, low vs. high GlycA levels before treatment significantly discriminated between low vs. high disease progression rate (δOsame/pulse time, Additional file 1: Fig. S1C, p = 0.021, Mann–Whitney test), while TNF levels did not. This finding, in addition to GlycA’s relative stability over time (Fig. 4C and [9–11]), underscores the potential of GlycA as a novel and clinically useful biomarker for corticosteroid response in HAM/TSP.

### Transcriptomic validation of cytokine signatures in PLHTLV-1 and incident HAM/TSP

**Fig. 5:**
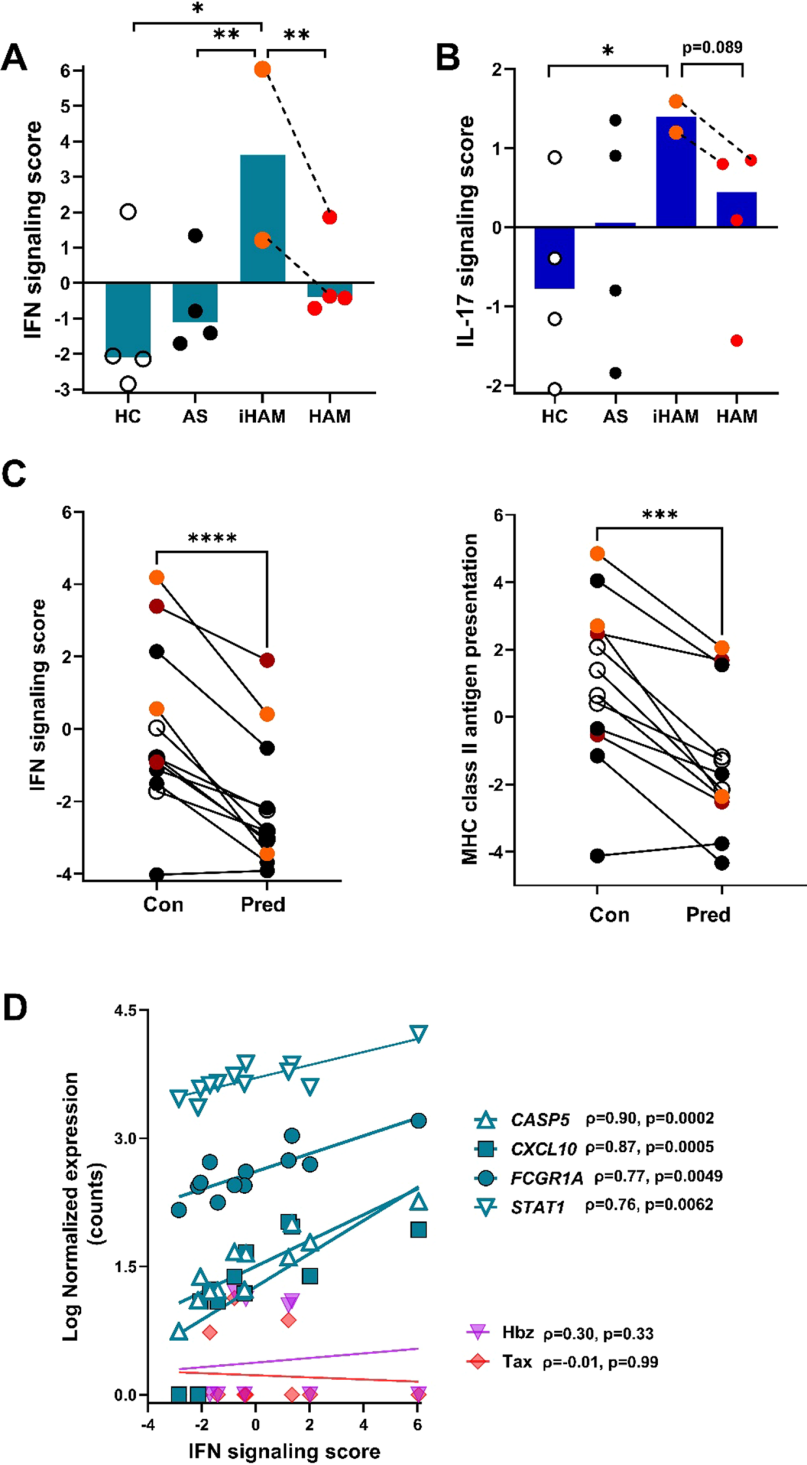
Transcriptomic validation of cytokine signaling pathways and prednisolone response in an independent cohort of PLHTLV-1. Digital transcriptomics (nCounter) was used to quantify cytokine signaling pathways in HAM/TSP disease progression, and the effect of in vitro prednisolone treatment in PBMCs from four AS, four HAM/TSP patients (including the two only incident HAM/TSP in the HOST cohort), and four age-, gender- and ethnicity-matched healthy controls. **A** Ex vivo IFN signaling score was significantly higher in incident HAM/TSP as compared to healthy controls, AS, and HAM/TSP at 4-year follow-up (one sample *t* test). **B** Ex vivo IL-17 signaling score was significantly higher in incident HAM/TSP as compared to healthy controls and tends to decline in HAM/TSP at 4-year follow-up (one sample *t* test). **C** IFN signaling was homogeneously down-regulated in all clinical groups by prednisolone treatment in vitro (left panel, Wilcoxon test *p* < 0.0001). Down-regulation was confirmed by decreased expression of the IFN-γ-regulated MHC Class II antigen presentation pathway (right panel, Wilcoxon test *p* < 0.001). **D** Ex vivo transcriptomic IFN signaling score measured by nCounter is significantly correlated with mRNA levels of previously identified HAM/TSP biomarkers *CASP5, FCGR1A, STAT1,* and *CXCL10* (all *p* < 0.05 with Bonferroni correction), but not to HTLV-1 mRNAs Hbz and Tax. HC: healthy controls (open circles); *AS* asymptomatics (black circles), *iHAM* incident HAM/TSP (orange circles), HAM/TSP at 4-year follow-up (red circles), *Con* untreated in vitro PBMCs, *Pred* prednisolone-treated PBMCs in vitro. Paired samples from iHAM patients at diagnosis and at follow-up are identified by dashed lines.

To confirm and extend our findings of cytokine signatures at the protein level (Figs. 1, 2, 3 and 4), we used digital transcriptomics (nCounter) to provide broader mechanistic insight into the downstream cytokine signaling pathways [20, 21] mediating HAM/TSP disease progression, and the effect of prednisolone treatment in vitro. From a well-characterized US cohort of PLHTLV-1 [22], we selected 4 AS and 4 HAM/TSP patients (including the only two incident HAM/TSP cases from the entire HTLV-1 cohort), and age-, gender- and ethnicity-matched healthy controls (*n* = 4). First, we confirm that exacerbated IFN signaling is a hallmark of HAM/TSP disease status [32], being significantly higher in incident HAM/TSP (Fig. 5A), as compared to healthy controls (fivefold, *p* = 0.022) and AS (fourfold p = 0.0085), when quantified ex vivo by nCounter. Likewise, ex vivo IL-17 signaling score was also increased in incident HAM/TSP (Fig. 5B), as compared to healthy controls (twofold, *p* = 0.044) and AS (1.5-fold, *p* = 0.14). Similar to our findings in HAM/TSP patients from the Brazilian cohort, IFN signaling (3.5-fold, *p* = 0.0096) and IL-17 signaling score (twofold, *p* = 0.089) were downregulated in the US cohort at (4-year) follow-up. However, no uniform HAM/TSP treatment protocol exists for the US HOST cohort, and the number of patients was too small to correlate cytokine signaling in vitro to therapeutic response or disability score.

Nevertheless, IFN signaling (measured by nCounter) was effectively and homogeneously down-regulated in all clinical groups by in vitro prednisolone treatment (Fig. 5C), which was further confirmed by decreased expression of the IFN-γ-regulated MHC Class II antigen presentation pathway (Fig. 5C). In contrast, in vitro prednisolone treatment did not have a uniform effect on IL-10 signaling, IL4/IL-13 signaling, IL-17 signaling or IL-2 cytokine family signaling (data not shown) but was strongly variable among the four clinical groups. Finally, we investigated if nCounter IFN signaling score was correlated with previously identified HAM/TSP transcriptomic biomarkers (*CASP5, FCGR1A, STAT1* identified in [32]) and *CXCL10*, proposed by several groups [4, 5, 8, 33] as a sensitive biomarker for HAM/TSP disease status and corticosteroid response. As shown in Fig. 5D, ex vivo* CASP5, CXCL10, FCGR1A* and *STAT1* transcript levels were significantly (all p < 0.05 with Bonferroni correction) correlated with IFN signaling score. In contrast, HTLV-1 transcripts Hbz and Tax were not significantly correlated with IFN signaling score (Fig. 5D), suggesting that different cytokine signatures in PLHLTV-1 and HAM/TSP patients are not directly triggered by retroviral transcription.

## Discussion

In this observational study, we selected 110 biobanked plasma samples, corresponding to all first available samples, i.e., closest to the date of entry in a large open cohort of PLHTLV-1, corresponding to a total of 946 person-years of clinical follow-up, including 43 HAM/TSP patients and 67 asymptomatic PLHTLV-1. Using these unique samples, we demonstrated systemic cytokines and GlycA as candidate biomarkers of inflammaging, immunopathogenesis and therapeutic response in HAM/TSP.

Inflammaging has been extensively documented in people living with HIV-1 [14, 15], but this is the first report of inflammaging in PLHTLV-1, characterized by an age-dependent increase in pro-inflammatory cytokine IL-6, which was positively correlated with chronic inflammation marker GlycA (Fig. 1). Among pro-inflammatory cytokines, IL-6 uniquely predicts global functional decline in aging [34] and inflammaging in a systematic review and meta-analysis [35]. With 110 PLHTLV-1 in our study, we had sufficient statistical power for a subgroup analysis. The significant (Bonferroni-corrected) correlation between age and systemic IL-6 levels in AS agrees with the extensively documented positive correlation in the general population. For instance, a recent study [18], described effect sizes for the correlation between age and IL-6 in two large Japanese cohorts (*ρ* = 0.44, *n* = 684 and *ρ* = 0.60, *n* = 841) that were very similar to ours (*ρ* = 0.50, *n* = 67 AS). On the other hand, the absence of correlation between pro-inflammatory IL-6 and age in HAM/TSP patients is a surprising new finding in (neuro)inflammatory disease, further strengthened by the absence of correlation between IL-6 and GlycA in HAM/TSP patients only. Again, the significant correlation between IL-6 and GlycA we observed in AS is similar to previous findings in a large populational study [10]. Moreover, both instances of loss of correlation in HAM/TSP patients were observed in pre-treatment samples, hence not biased by pharmacological intervention and likely to reflect an early feature of HAM/TSP pathogenesis.

Although IL-10 was weakly correlated with age in asymptomatics, the IL6/IL10 ratio, representing a higher pro-inflammatory state, significantly increases with age (± 50%/15 years, *p* = 0.014), and also corroborates the increased mortality rate observed in this cohort [7], as well as in a recent systematic review and meta-analysis of HTLV-1 adverse health outcomes [36]. Of note, in addition to its widely documented anti-inflammatory role, IL-10 might also exert pro-inflammatory functions, especially in the context of type I IFN, as reviewed by Mühl [37]. Since the IL6/IL10 ratio has been demonstrated as a sensitive biomarker of COVID-19 outcome [38], aging PLHTLV-1 might be at increased risk of developing severe or critical COVID-19. Of interest, this hypothesis was also supported by in silico findings [39]. Furthermore, we have previously described an age-dependent decrease of B cells in HAM/TSP patients [26], which might also lead to decreased SARS-CoV-2 vaccine-induced antibody response. Both hypotheses obviously remain to be confirmed in large observational studies, which are challenging in neglected diseases [40].

Regarding disease status, we have used complimentary analytical approaches, namely, multivariable regression, machine learning-derived decision trees and Bayesian network learning to model cytokine and GlycA interactions with demographic and clinical parameters. While multivariable logistic regression identified IL-17A and proviral load as independent predictors of HAM/TSP disease status, Bayesian network analysis enables visualization of the co-dependencies between cytokines in PLHTLV-1, and their associations with GlycA and disease status. Thus, we found that IL-17A appears intrinsically related to disease status in all three analytical models (regression, decision tree and Bayesian network). However, IFN-γ appears “upstream” of all other cytokines in the Bayesian network, which corroborates previous transcriptomic findings [32]. This study confirms the findings of Kagdi et al. for IFN-γ and IL-17A as biomarkers of untreated HAM/TSP [28]. In spite of our larger cohort (67 AS, 43 HAM/TSP), we did not observe increased IL-2 nor IL-10 in HAM/TSP, in contrast to Kagdi et al. (17 AS, 28 HAM/TSP). However, our decision tree (Fig. 3A) identified by Machine Learning is quite similar to the decision tree proposed by Kagdi et al. to classify AS, HAM/TSP and ATL patients based upon IL-10 and IL-17 levels. Exacerbated IFN-γ production has been consistently demonstrated by numerous groups, either ex vivo (in serum/plasma) or in vitro (in supernatants of PBMCs cultured for 1–4 days), as a hallmark of HAM/TSP [4, 5, 28, 41–45]. Other pro- and anti-inflammatory cytokines (IL-2, IL-4, IL-6, IL-10, IL-17A, TNF) have yielded strongly diverging results between different groups regarding their up- or down-regulation in HAM/TSP vs. AS [28, 41–48]. This is most likely due to smaller cohort sizes, in addition to the choice of plasma/serum vs. cell culture supernatants or intracellular flow cytometry. In addition, differences in age, gender, genetics as well as the inclusion of treated HAM/TSP patients in some cohorts might also explain the observed discrepancies. Indeed, we found that IFN-γ and IL-17A are differentially impacted by corticosteroid pulse therapy: post-treatment IFN-γ levels are low in responders, while IL-17A levels decrease uniformly for all patients. Current clinical guidelines for HAM/TSP suggest that early HAM/TSP patients might benefit most from corticosteroid therapy [6], which is also supported the recent (and first placebo-controlled) randomized clinical trial for corticosteroid therapy in HAM/TSP (HAMLET-P [33]).

In addition to IL-17A, we found proviral load is an independent biomarker of untreated disease in HAM/TSP patients, consenting with the literature [4–7]. However, proviral load did not predict incident HAM/TSP cases in three out of four published Brazilian cohort studies [49–52]. Of those, only Tanajura et al. demonstrated proviral load as a significant predictor of neurological symptoms, but not definite HAM/TSP, during clinical follow-up [50]. Similar to Yamauchi et al. [8], we found that proviral load is not a biomarker for therapeutic response in HAM/TSP. However, we identified, for the first time, TNF and GlycA as independent predictors of clinical worsening, as measured by increased Osame Motor Disability Scale. To put this prediction into a patient-centered clinical context, the median Osame Motor Disability Scale of 4 in the low TNF/GlycA group corresponds to “needs a handrail when climbing stairs”, whereas the median of 8 in the high TNF/GlycA group corresponds to “can walk 1-5 m with bilateral support”. Notably, the high TNF/GlycA group also comprised the only fatal case among 43 HAM/TSP patients, with death related to HAM/TSP as described in our previous study [7]. Several other non-cytokine biomarkers, such as CXCL10 (also known as IP-10), Neurofilament Light Chain (NFL), and Chitotriosidase-1 (encoded by the *CHIT1* gene) have recently been proposed as biomarkers for HAM/TSP disease progression [4, 5, 8, 33, 53–58]. Although most inflammatory biomarkers correlate reasonably well between plasma and CSF [53–59], most of these published studies have focused on CSF samples for the quantification of these biomarkers, which was neither logistically nor ethically possible in our cohort. Moreover, our goal was to define biomarkers that can be easily implemented in clinical practice, such as non-invasive plasma/serum samples, which also allow repeated testing before and after treatment at regular intervals. Thus, we quantified plasma CXCL10 protein levels by Bio-Plex immunoassay in 41 individuals from the São Paulo cohort (41 out of 110 PLHTLV-1 with sufficient samples). We found that CXCL10 plasma levels did not differ significantly between AS (median 37,164 pg/ml, IQR [16,946–69,585], *n* = 26) and HAM/TSP patients (before treatment, median 38,631, IQR [19,465–69,723], *n* = 15). In addition, CXCL10 levels did not significantly correlate to age at sampling, sex or proviral load (all *p* > 0.05, not shown). CXCL10 plasma levels also did not correlate to disease duration, disease severity (Osame Motor Scale), age of onset or therapeutic response to methylprednisolone pulse therapy (all *p* > 0.05, not shown). However, these results should be interpreted with caution, due to the low number of HAM/TSP patients in this subset analysis. Finally, CXCL10 plasma levels were not significantly correlated with GlycA levels, neither with any of the 7 plasma cytokines (after Bonferroni correction).

Notable strengths and limitations of this study merit further detail. First, this study has a relatively large sample size, to our knowledge the largest yet with regard to plasma cytokines as candidate biomarkers in PLHTLV-1 and HAM/TSP. Second, complete neurological evaluations of PLHTLV-1 and uniform treatment strategy for HAM/TSP patients are major strengths, as well as a remarkably long follow-up (median 8.6 years in Brazil cohort, > 14 years in US cohort). A major limitation is the low incidence in the US cohort (2/2100 person-years), thus limiting our statistical power for replication. Other limitations include the lack of simultaneous protein (plasma/serum, CSF) and RNA (PBMC, CSF) quantification in both cohorts due to sample unavailability, as well as potential selection biases regarding patient recruitment and loss to follow-up [7], which are inherent to cohort studies in neglected diseases.

## Conclusions

We found that the untreated disease status in HAM/TSP patients, as compared to age- and gender-matched asymptomatic PLHTLV-1, is characterized by increased systemic IFN-γ and IL-17A. From a clinical point of view, plasma GlycA, IL-6, TNF and IFN-γ are promising candidate biomarkers for immunomonitoring of inflammaging in PLHTLV-1, and of disease progression and corticosteroid therapeutic response in HAM/TSP patients, respectively. In addition, we provide predictive regression models and decision trees for prospective testing in clinical trials or independent cohort studies.

## Supplementary Information

**Additional file 1: Table S1.** Multivariable logistic regression (Asymptomatics (AS) vs. HAM/TSP patients). **Table S2.** Multivariable linear regression of disease progression (measured by Osame Motor Disability Scale) in HAM/TSP patients. **Fig S1.** GlycA pre-treatment levels predict disease progression rate under prednisolone pulse therapy, independent of age at onset or disease duration.

## Data Availability

All data contained in the manuscript are available from the corresponding authors.

## Abbreviations

AS: Asymptomatics
HAM/TSP: HTLV-1-Associated Myelopathy/Tropical Spastic Paraparesis
HIV: Human immunodeficiency virus
HTLV: Human T-cell leukemia virus
IFN: Interferon
IL: Interleukin
PLHTLV-1: People living with HTLV-1
TNF: Tumor Necrosis Factor
