# Supplementary material for: Systemic cytokines and GlycA discriminate disease status and predict corticosteroid response in HTLV-1-associated neuroinflammation
Source: J Neuroinflammation. 2022 Dec 8;19:293. doi: 10.1186/s12974-022-02658-w (PMC9733207; doi:10.1186/s12974-022-02658-w)
Supplement: Supplementary file 1 — Additional file 1: Table S1. Multivariable logistic regression (Asymptomatics (AS) vs. HAM/TSP patients). Table S2. Multivariable linear regression of disease progression (measured by Osame Motor Disability Scale) in HAM/TSP patients. Fig S1. GlycA pre-treatment levels predict disease progression rate under prednisolone pulse therapy, independent of age at onset or disease duration. [file 12974_2022_2658_MOESM1_ESM.docx]

**Additional file 1: Table S1. Multivariable logistic regression (Asymptomatics (AS) vs. HAM/TSP patients)**

| **Model 1** |  |  |  |  |
| --- | --- | --- | --- | --- |
| **Parameter estimates** | **Variable** | **Estimate** | **Standard error** | **95% CI** |
| β0 | **Intercept** | -5.607 | 1.911 | -9.758 to -2.213 |
| β1 | **Sex** | 0.5821 | 0.5803 | -0.5570 to 1.740 |
| β2 | **Age sampling** | -0.021 | 0.01928 | -0.06009 to 0.01646 |
| β3 | **log PVL** | 0.9479 | 0.2756 | 0.4702 to 1.561 |
| β4 | **IL-17A** | 0.0279 | 0.009137 | 0.01207 to 0.04782 |
|  |  |  |  |  |
| **Odds ratios** | **Variable** | **Estimate** | **95% CI** |  |
| β0 | Intercept | 0.003671 | 5.782e-005 to 0.1094 |  |
| β1 | Sex | 1.79 | 0.5729 to 5.699 |  |
| β2 | Age sampling | 0.9792 | 0.9417 to 1.017 |  |
| β3 | log PVL | 2.58 | 1.600 to 4.765 |  |
| β4 | IL-17A | 1.028 | 1.012 to 1.049 |  |
|  |  |  |  |  |
| **Area under the ROC curve** |  |  |  |  |
| Area | 0.8525 |  |  |  |
| Std. Error | 0.04051 |  |  |  |
| 95% confidence interval | 0.7731 to 0.9318 |  |  |  |
| P value | <0.0001 |  |  |  |
|  |  |  |  |  |
| **Classification table** | **Predicted 0** | **Predicted 1** | **Total** | **% Correctly classified** |
| Observed AS | 51 | 6 | 57 | 89.47 |
| Observed HAM/TSP | 12 | 27 | 39 | 69.23 |
| Total | 63 | 33 | 96 | 81.25 |
|  |  |  |  |  |
| **Model 2** |  |  |  |  |
| **Parameter estimates** | **Variable** | **Estimate** | **Standard error** | **95% CI** |
| β0 | **Intercept** | -6.619 | 1.526 | -10.02 to -3.985 |
| β1 | **log PVL** | 0.9814 | 0.2739 | 0.5038 to 1.590 |
| β2 | **IL-17A** | 0.02763 | 0.008742 | 0.01243 to 0.04664 |
|  |  |  |  |  |
| **Odds ratios** | **Variable** | **Estimate** | **95% CI** |  |
| β0 | Intercept | 0.001335 | 4.450e-005 to 0.01859 |  |
| β1 | log PVL | 2.668 | 1.655 to 4.903 |  |
| β2 | IL-17A | 1.028 | 1.013 to 1.048 |  |
|  |  |  |  |  |
| **Area under the ROC curve** |  |  |  |  |
| Area | 0.8538 |  |  |  |
| Std. Error | 0.04077 |  |  |  |
| 95% confidence interval | 0.7739 to 0.9337 |  |  |  |
| P value | <0.0001 |  |  |  |
|  |  |  |  |  |
| **Classification table** | **Predicted 0** | **Predicted 1** | **Total** | **% Correctly classified** |
| Observed AS | 49 | 8 | 57 | 85.96 |
| Observed HAM/TSP | 14 | 25 | 39 | 64.1 |
| Total | 63 | 33 | 96 | 77.08 |
|  |  |  |  |  |
| **Comparison of models** |  |  |  |  |
| Simpler model | Model 2 |  |  |  |
| Probability it is correct | 77.12% |  |  |  |
| Alternative model | Model 1 |  |  |  |
| Probability it is correct | 22.88% |  |  |  |
| Ratio of probabilities | 3.371 |  |  |  |
| **Preferred model*** | **Model 2** |  |  |  |
| Difference in AICc | -2.430 |  |  |  |
|  |  |  |  |  |
|  |  |  |  |  |
| **Data summary** |  |  |  |  |
| Number of individuals | 110 |  |  |  |
| Missing data (proviral load, PVL) | 14 |  |  |  |
| Rows analyzed (#observations) | 96 |  |  |  |
| Number of HAM/TSP | 39 |  |  |  |
| Number of AS | 57 |  |  |  |
| Number of parameter estimates | 5 |  |  |  |
| #observations/ #parameters | 19.2 |  |  |  |
| # of HAM/TSP/#parameters | 7.8 |  |  |  |
| # of AS/#parameters | 11.4 |  |  |  |

***Best model selected by corrected Akaike’s Information Criterion (AICc)**

**Additional file 1: Table S2. Multivariable linear regression of disease progression (measured by Osame Motor Disability Scale) in HAM/TSP patients**

| **Model 1** |  |  |  |  |
| --- | --- | --- | --- | --- |
| **Parameter estimates** | **Variable** | **Estimate** | **Standard error** | **95% CI** |
| **β0** | **Intercept** | **-7.083** | **2.672** | **-12.60 to -1.567** |
| β1 | Sex | -0.02114 | 0.9482 | -1.978 to 1.936 |
| β2 | Age sampling | -0.005281 | 0.03473 | -0.07696 to 0.06640 |
| β3 | TNF | 0.02472 | 0.01398 | -0.004143 to 0.05358 |
| **β4** | **GlycA** | **9.292** | **2.715** | **3.689 to 14.90** |
|  |  |  |  |  |
| **Multicollinearity** | **Variable** | **VIF** | **R2 with other variables** |  |
| β0 | Intercept |  |  |  |
| β1 | Sex | 1.328 | 0.2468 |  |
| β2 | Age sampling | 1.219 | 0.1798 |  |
| β3 | TNF | 1.236 | 0.1907 |  |
| β4 | GlycA | 1.297 | 0.2293 |  |
|  |  |  |  |  |
| **Model 2** |  |  |  |  |
| **Parameter estimates** | **Variable** | **Estimate** | **Standard error** | **95% CI** |
| **β0** | **Intercept** | **-7.293** | **2.198** | **-11.81 to -2.774** |
| **β1** | **TNF** | **0.02545** | **0.01210** | **0.0005791 to 0.05032** |
| **β2** | **GlycA** | **9.182** | **2.292** | **4.471 to 13.89** |
|  |  |  |  |  |
| **Multicollinearity** | **Variable** | **VIF** | **R2 with other variables** |  |
| β0 | Intercept |  |  |  |
| β1 | TNF | 1.001 | 0.0009555 |  |
| β2 | GlycA | 1.001 | 0.0009555 |  |
|  |  |  |  |  |
| **Comparison of models** |  |  |  |  |
| Simpler model | Model 2 |  |  |  |
| Probability it is correct | 95.53% |  |  |  |
| Alternative model | Model 1 |  |  |  |
| Probability it is correct | 4.471% |  |  |  |
| Ratio of probabilities | 21.36 |  |  |  |
| **Preferred model*** | **Model 2** |  |  |  |
|  |  |  |  |  |
|  |  |  |  |  |
| **Data summary** |  |  |  |  |
| Rows in table | 35 |  |  |  |
| Rows skipped (missing data) | 6 |  |  |  |
| Rows analyzed (# cases) | 29 |  |  |  |
| Number of parameter estimates | 5 |  |  |  |
| #cases/#parameters | 5.8 |  |  |  |

***Best model selected by corrected Akaike’s Information Criterion (AICc)**


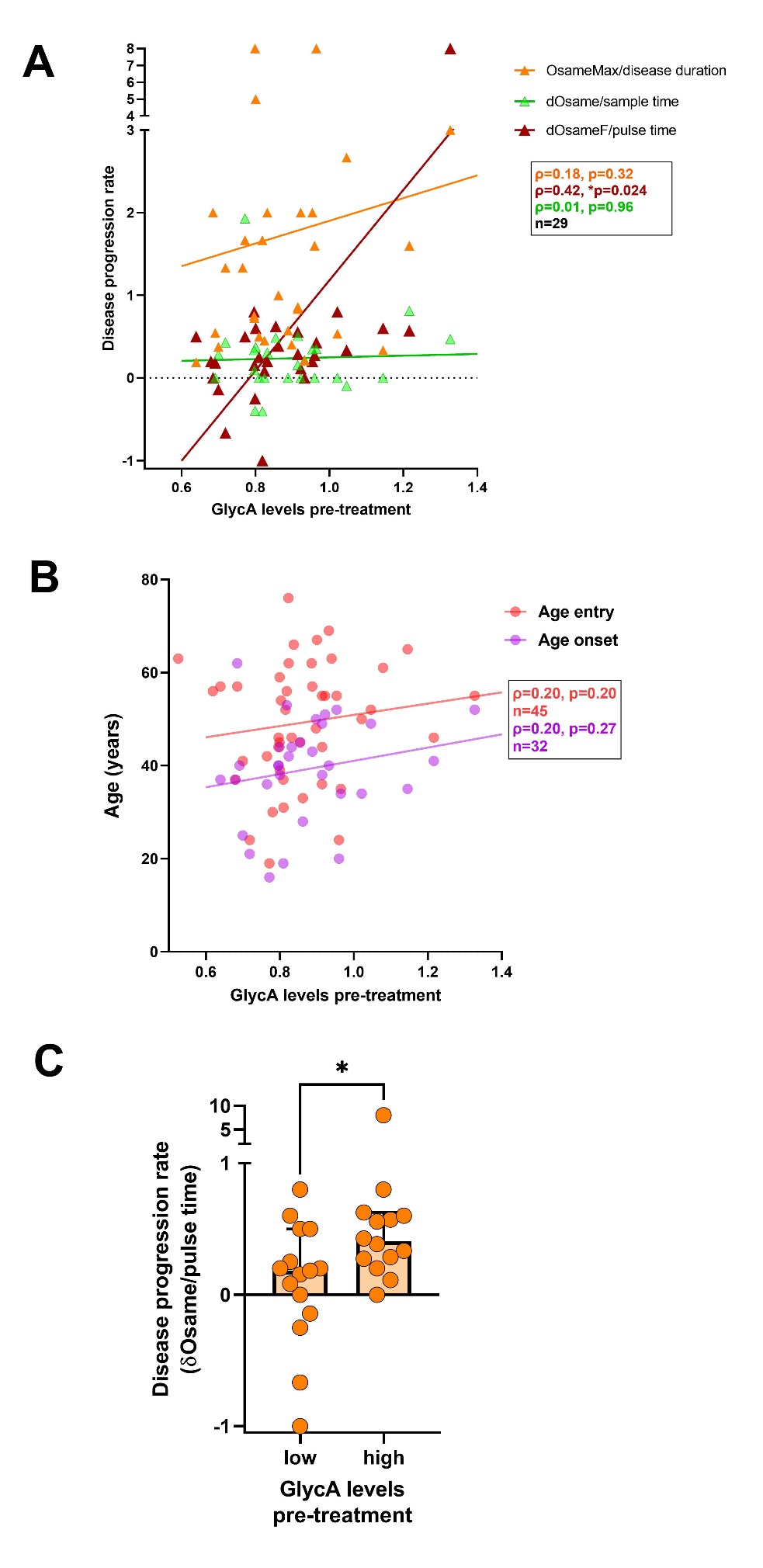


**Additional file 1: Figure S1. GlycA pre-treatment levels predict disease progression rate under prednisolone pulse therapy, independent of age at onset or disease duration**

(A) Pre-treatment GlycA levels are correlated with the disease progression rate calculated over pulse time (δOsame/pulse time = quantitative change in Osame Motor Disability Score/total pulse time) but not with disease progression rate calculated over total clinical follow-up (δOsame/sample time = quantitative change in Osame Motor Disability Score/total time of clinical follow-up at sampling), nor maximal Osame Motor Disability Score/total disease duration. Spearman correlations are shown. (B) Pre-treatment GlycA levels are not significantly correlated with age of onset or age at entry in the cohort (Spearman correlations are shown). (C) Low vs. high (above or below the median) pre-treatment GlycA levels predict lower vs. higher disease progression rate after corticosteroid pulse therapy (Wilcoxon test, p=0.021).
